# Supplementary material for: Analysis of PPARγ Signaling Activity in Psoriasis
Source: Int J Mol Sci. 2021 Aug 10;22(16):8603. doi: 10.3390/ijms22168603 (PMC8395241; doi:10.3390/ijms22168603)
Supplement: Supplementary file 1 [file ijms-22-08603-s001.zip › Supplemental materials_Analysis of PPARg signaling activity in psoriasis/Pathway models/Models images and html files/Interleukin-17 and interleukin-22 signaling in psoriasis/10229.html]

S100A7


# Protein S100A7

|  |  |
| --- | --- |
| URN | urn:agi-llid:6278 |
| Total Entities | 0 |
| Connectivity | 858 |
| Name | S100A7 |
| Description | S100 calcium binding protein A7 |
| Notes | The protein encoded by this gene is a member of the S100 family of proteins containing 2 EF-hand calcium-binding motifs. S100 proteins are localized in the cytoplasm and/or nucleus of a wide range of cells, and involved in the regulation of a number of cellular processes such as cell cycle progression and differentiation. S100 genes include at least 13 members which are located as a cluster on chromosome 1q21. This protein differs from the other S100 proteins of known structure in its lack of calcium binding ability in one EF-hand at the N-terminus. The protein is overexpressed in hyperproliferative skin diseases, exhibits antimicrobial activities against bacteria and induces immunomodulatory activities. [provided by RefSeq, Nov 2014] |
| Primary Cell Localization | Plasma membrane |

---

|  |  |
| --- | --- |
| Pathway | Proteins Involved in Atherosclerosis |
|  | IL17 Signaling in Psoriasis |
|  | Proteins Involved in Psoriasis |
|  | IL22 Induces Keratinocyte Proliferation in Psoriasis |
|  | Proteins with Altered Expression in Psoriasis |
|  | Dendritic Cells Function in Psoriasis |
|  | Keratinocyte Activation in Psoriatic Arthritis |
|  | Overview. Diseases of the skin (chapter 11) |
|  | 2\_Interleukin-17 and interleukin-22 signaling in psoriasis |
|  | Interleukin-17 and interleukin-22 signaling in psoriasis |

---

|  |  |
| --- | --- |
| MedScan ID | 6278 |

---

|  |  |
| --- | --- |
| LocusLink ID | 6278 |

---

|  |  |
| --- | --- |
| Alias | Psoriasin |
|  | OTTHUMP00000015328 |
|  | PSOR1 |
|  | PSOR I |
|  | psoriasin 1 |
|  | OTTHUMP00000015327 |
|  | S100RVP |
|  | psoriasin I |
|  | S100 calcium binding protein A7 |
|  | S100A7 |
|  | s100a7c |
|  | S100 calcium binding protein A7 (psoriasin 1) |
|  | S100 calcium-binding protein |
|  | LOC310568 |
|  | protein S100-A7 |
|  | S100 calcium-binding protein A7 (psoriasin 1) |

---

|  |  |
| --- | --- |
| GO ID | 0050786 |
|  | 0005509 |
|  | 0008270 |
|  | 0001525 |
|  | 0061844 |
|  | 0019730 |
|  | 0050829 |
|  | 0008544 |
|  | 0045087 |
|  | 0030216 |
|  | 0043312 |
|  | 0070374 |
|  | 0010820 |
|  | 0071624 |
|  | 0090026 |
|  | 0032496 |
|  | 0000302 |
|  | 0051238 |
|  | 0035578 |
|  | 0062023 |
|  | 0005737 |
|  | 0005829 |
|  | 0005783 |
|  | 0005576 |
|  | 0005615 |
|  | 0005925 |
|  | 0005634 |
|  | 0070062 |

---

|  |  |
| --- | --- |
| KEGG ID | hsa:6278 |

---

|  |  |
| --- | --- |
| Organism | Homo sapiens {Organism urn:agi-taxid:9606} |
|  | Homo sapiens |

---

|  |  |
| --- | --- |
| OMIM ID | 600353 |

---

|  |  |
| --- | --- |
| Hugo ID | 10497 |

---

|  |  |
| --- | --- |
| Human chromosome position | 1q21.3 |
|  | 1q21 |

---

|  |  |
| --- | --- |
| Swiss-Prot Accession | P31151 |
|  | P31151.4 |
|  | Q5SY67 |
|  | Q6FGE3 |
|  | Q9H1E2 |

---

|  |  |
| --- | --- |
| PIR ID | A54327 |

---

|  |  |
| --- | --- |
| GenBank ID | NC\_000001 |
|  | NM\_002963 |
|  | NP\_002954 |
|  | AF050167 |
|  | AJ012825 |
|  | CAC20409 |
|  | AL591704 |
|  | BR000043 |
|  | FAA00017 |
|  | CH471121 |
|  | EAW53327 |
|  | AW238300 |
|  | BC034687 |
|  | AAH34687 |
|  | BF090388 |
|  | BX476368 |
|  | CR542164 |
|  | CAG46961 |
|  | M86757 |
|  | AAA60210 |
|  | S81991 |
|  | P31151 |
|  | NC\_018912 |
|  | AMYH02001902 |
|  | DQ891277 |
|  | ABM82203 |
|  | DQ894465 |
|  | ABM85391 |

---

|  |  |
| --- | --- |
| Swiss-Prot ID | S10A7\_HUMAN |

---

|  |  |
| --- | --- |
| Cell Localization | Cytoplasm |
|  | Secreted |

---

|  |  |
| --- | --- |
| Ensembl ID | ENSG00000143556 |
|  | ENSP00000357712.3 |
|  | ENST00000368723.4 |
|  | ENSP00000357712 |
|  | ENST00000368723 |

---

|  |  |
| --- | --- |
| Unigene ID | Hs.112408 |

---
